# The multifaceted challenge of evaluating protected area effectiveness

**DOI:** 10.1038/s41467-020-18989-2

**Published:** 2020-10-13

**Authors:** Ana S. L. Rodrigues, Victor Cazalis

**Affiliations:** CEFE, Univ. Montpellier, CNRS, EPHE, IRD, Univ. Paul Valéry Montpellier 3, Montpellier, France

**Keywords:** Conservation biology, Biodiversity

## Abstract

Protected areas (PAs) are the most important conservation tool, yet assessing their effectiveness is remarkably challenging. We clarify the links between the many facets of PA effectiveness, from evaluating the means, to analysing the mechanisms, to directly measuring biodiversity outcomes.

The modern movement to designate protected areas (PAs) started in the mid-1800s, even if the drive to protect ‘special places’, such as sacred grounds or hunting areas, goes back millennia^1^. If the purpose of the early PAs was to safeguard beautiful landscapes or to control access to valuable natural resources^1^, today’s PAs are much more than a collection of exceptional sites. They are the main conservation tool on which hopes for stemming the relentless decline in the global biodiversity largely rest.

This vision of PAs as an international cooperation endeavour goes back to the early years of the International Union for Conservation of Nature (IUCN), through the work of its World Commission on Protected Areas (WCPA), created in 1960 (ref. ^1^). It was consolidated in the 1992 United National Convention on Biological Diversity (CBD), which mandates each contracting party to establish a system of PAs^2^. PAs remain central international conservation policy, including under the CBD 2011–2020 Strategic Plan for Biodiversity^3^, and post-2020 Global Biodiversity Framework^4^.

Measured by extension alone, PAs are an extraordinarily successful conservation response. Found in all countries, they currently occupy 15.0% of the global land surface and 7.4% of the world’s oceans^5^. Their coverage has been expanding steadily^5^, and will likely continue to do so in response to increasingly ambitious policy targets^4^.

Confidence on PAs as a conservation tool makes good sense. Indeed, they can counter habitat loss and degradation through restrictions to activities, such as agriculture, deforestation, urbanisation, mining and road construction, by far the major causes of biodiversity decline worldwide^6^, as well as locally attenuate pressures, such as overexploitation, pollution or invasive species. However, PAs are not all the same. Indeed, they vary substantially in the nature of intended management, from sites strictly protected for biodiversity, to multiple-use areas involving natural resource use, such as forestry or hunting^5^. They also vary—perhaps even more so—in the extent to which their intended management translates into practical reinforcement, from sites with legal existence but no on-the-ground presence, to areas benefiting from substantial staff and funding^7^. Other factors affect the extent to which PAs can fulfil their conservation role. For example, they may be too small, too isolated, or too few to counter the surrounding pressures, or placed in locations where their value as conservation tools is limited. Consequently, the effectiveness of PAs as conservation measures, individually as well as collectively, cannot be taken for granted.

## Defining protected area effectiveness

A PA is ‘a clearly defined geographical space, recognised, dedicated and managed, through legal or other effective means, to achieve the long-term conservation of nature with associated ecosystem services and cultural values’^5^. Perfectly effective PAs would thus ‘achieve’ the long-term conservation of nature, at least within their boundaries, ideally beyond by working as an effective network. In practice, though, effectiveness is not a binary yes-or-no trait, but a multifaceted gradient measuring the extent to which PAs contribute to conservation outcomes.

## The means, the mechanisms and the ends

**Fig. 1:**
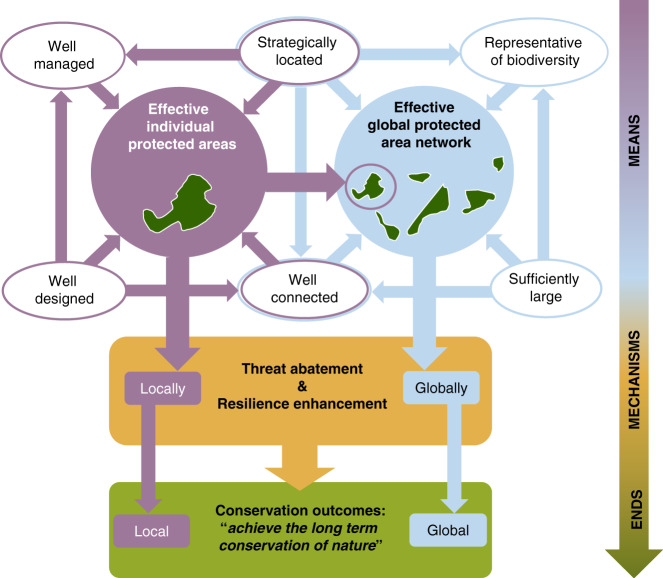
The many facets of protected area effectiveness, from the means to the mechanisms, to the ends. Arrows indicate the direction of effects between elements (e.g., strategic location impacts representativeness).

The effectiveness of individual PAs arises from a set of intertwined factors (Fig. 1), including both decisions taken at the time of establishment (design, location and connectivity to other sites), as well as subsequent management decisions. The effectiveness of the global network is in turn determined by the effectiveness of its individual components and by how they relate to each other (in total extent, location, connectivity and representativeness). Each of these individual factors is not a conservation end in its own right, but a means for achieving the conservation of nature. The means translate into conservation outcomes through two mechanisms: threat abatement through effective management; and resilience enhancement through location and design factors determining the capacity of PAs (individually and collectively) to conserve genetic, specific and ecosystem diversity over the long-term.

Most previous analyses of PA effectiveness focus on the means of protection, in particular, the total extent of the network, management inputs and location considerations^5,7,8^. Many of these studies are in the context of measuring progress towards Aichi Target 11 of the 2011–2020 CBD Strategic Plan for Biodiversity^3^, which included quantitative targets for extent (17% of terrestrial area and 10% of the oceans by 2020), and qualitative targets for location (focus on ‘areas of particular importance for biodiversity’; ‘ecologically representative and well connected systems’) and for management (‘effectively and equitably managed PAs’). Recent studies have also investigated the effects of PAs on threat abatement (mainly on habitat loss)^9,10^, with a smaller but increasing number of analyses attempting to quantify conservation outcomes directly^11–14^.

## Evaluating extent

From its inception, the movement for the establishment of PAs focused on expanding their coverage^1^. Evaluating total extent, however, requires a global inventory of sites, based on agreed criteria about what constitutes a PA. The world’s official inventory is the United Nations List of Protected Areas, first published in 1963. In the early 2000, with the advent of Geographic Information Systems and the internet, this list evolved towards the spatially explicit, publicly available World Database on Protected Areas (WDPA)^5^. Currently including 258,389 designated sites^5^, the WDPA rendered it straightforward to monitor progress in PA extent, including not only PA creation but also downsizing and degazettement^15^. This facet of PA effectiveness has taken a central place in conservation policy targets: Aichi Target 11 aimed for the protection of 17% of terrestrial area and 10% of the oceans by 2020 (not met^5^), and an even more ambitious 30% target for both land and seas is anticipated under the post-2020 Global Biodiversity Framework^4^. These targets consider not only PAs, but also other effective area-based conservation measures, a complementary approach to PAs that is still being formalised^16^. Focusing on targets on PA extent can however have perverse outcomes if it encourages nations to expand protection towards areas of little conservation value, or without the necessary subsequent investment in management^17^.

## Evaluating management

Possibly as old as PAs themselves, is the concern that they may not be up to expectations. PAs vary in purpose (as reflected in IUCN’s PAs management categories, going back to 1978 (ref. ^1^)), but there may in addition be a mismatch between the intended outcomes and what is achieved through management. A 2000 WCPA framework set the conceptual basis for assessing the effectiveness of PAs^18^, triggering the development of a diversity of methodologies usually involving questionnaires to PA managers to evaluate management elements of planning, inputs, processes, outputs and outcomes^7^. Over 55,000 such evaluations have already been completed and are available through the Global Database on Protected Area Management Effectiveness^19^. Analysis of a subset of these evaluations revealed that less than a quarter of PAs reported had adequate resources in terms of staffing and budget^7^.

## Evaluating location

PAs can only be expected to protect the biodiversity features they cover in the first place, yet in many parts of the world they are biased towards areas of little economic value, rather than placed strategically for meeting conservation targets^20^. Evaluating the representativeness of the global PA network requires crossing the location of protected sites with the distribution of the biodiversity features of interest. Such analyses only became possible at the global scale two decades ago, with the development of the WDPA and the comprehensive mapping of species across entire taxa (initially: birds, amphibians and mammals), as part of IUCN Red List assessments^6^. Crossing between the two revealed major gaps in the global PA network^21^ which, despite some progress, still remain: as of 2020, more than one in two terrestrial ecoregions had not met the 17% target, and 39% of sites identified as Key Biodiversity Areas had no protection at all^5^.

## Evaluating threat abatement

Many of the world’s PAs have ongoing and increasing human pressures^9^, but that does not mean they are not making a difference to abating threats. Investigating this requires contrasting observed threat levels with a counterfactual scenario of ‘what would have happened in the absence of protection’. Such counterfactual thinking has only recently started being applied to evaluating the effectiveness of conservation interventions^22^. With experimentation not feasible at the scale of PAs, quasi-experimental designs are used instead, whereby counterfactuals are derived statistically by contrasting PAs with suitable control, non-PAs. For some types of habitat loss and degradation, suitable datasets can be obtained from high-resolution satellite imagery (e.g., on forest cover change over time) showing, for example, that on average, PAs reduce pressures to terrestrial habitats, as seen by lower deforestation rates than in comparable non-protected sites^10^. However, for many types of threats (e.g. overexploitation, invasive species), the necessary data must come from field surveys, and for now large scale evaluations of effectiveness have not yet been possible.

## Evaluating biodiversity outcomes

A counterfactual approach is also needed for evaluating biodiversity outcomes. Again, fine-scale field data are needed, on the distribution of the biodiversity features of interest from both PAs and non-PAs. Suitable datasets are scarce, and highly biased towards high-income nations and better-known taxa (particularly birds). Improving such datasets, particularly in biodiversity-rich tropical regions, is thus crucial to outcome-based evaluations of PA effectiveness.

Analysing biodiversity outcomes also requires formalising the facets of biodiversity that PAs are expected to impact^12^. Among the few available studies, some report a positive effect of protection on local species richness and overall abundance^11,23^. Others however found no effect on species richness, but a positive effect of some specific groups, like specialist^12,14^, vulnerable^13,14^ or narrow-range species^14^, consistent with an effect of PAs in avoiding community homogenisation. Given the poor sensitivity of local species richness to anthropogenic land use transformation^24^, it is arguably a poor indicator of PA effectiveness, which should instead focus on effects on the species that need conservation the most^12,14^.

Previous analyses of biodiversity outcomes measured the average local effect of individual sites, not the extent to which PAs as a whole contribute to the global-scale conservation of nature. The latter is not the simple sum of individual effects, because of synergies between individual sites (e.g., through meta-population connectivity), as well as spillovers between protected and non-PAs, either positive (e.g., PAs acting as population sources) or negative (e.g., leakage of deforestation avoided within PAs into the surroundings). A major challenge in the evaluation of PA effectiveness is thus to scale up from the average individual sites, to quantifying the contribution of the overall PA network to global conservation outcomes.

## Conclusions

Whereas there is overwhelming evidence that the means currently allocated to PAs are insufficient, it remains unclear the extent to which these conservation tools are already contributing to the long-term conservation of nature, individually as well as globally. A stronger focus is thus needed on evaluating PA effectiveness in terms of biodiversity outcomes^11–13,23^, as well as on the links between the means and the outcomes of protection^23,25^, in order to better guide future conservation policy and practice.
